# Lessons Learnt and the Need to Apply Them

**Published:** 1919-06-14

**Authors:** 


### PUBLIC HEALTH AND THE WAR.

### Lessons Learnt and the Need to Apply Them.

In the early days of 1914 an old Army Chaplain, who had served all through the South African War, said "No man goes through a war and comes out of it unaltered." This was a true saying: it was true of the men themselves, and also of all the varied and complex relations between individuals that make up our modern manner of living.

In nothing will the changes effected be more important and more far-reaching than in our Public Health. It would be interesting—if it were possible—to know just what this term "Public Health" conveyed to the average lay mind before the War. To many it was inseparably connected with that long-suffering and sadly misnamed individual—the "Inspector of Nuisances." To some it was synonymous with a corporation of busybodies, who pursued inquisitive and sometimes awkward inquiries into such matters as the cleanliness of school children, the ventilation of workrooms, the proper stoking of furnaces, and the notification of consumption and other infectious diseases. Of course, there were some who knew better, but there were very few who appreciated the tremendous possibilities included in the term, and fewer still who dreamed that those possibilities would so soon come within the range of practical politics.

### Sanitation in the Field.

The effect of four years of war upon our outlook in these matters has been felt through every grade of society. Let us consider first its influence on those who have served in His Majesty's Forces by sea, land, or air. To soldiers, probably more than anyone else, has the true meaning and use of sanitation been brought home. Both officers and men have certainly been lectured to and taught more about hygiene than ever before. But—what has been far more useful—they have seen for themselves the effects of neglecting the rules laid down for the maintenance of health. To give one instance, many an officer and man has seen the result of a cook being dirty, in his person or habits, in the subsequent illness of his mess-mates or himself. They have learned also to appreciate the importance of having a pure water supply, the meaning and need for conservancy, and the reason for properly disposing of greasy water and refuse. All these officers and men will bring this knowledge and experience home with them; they will take a more intelligent interest in public affairs; the news that the Town or Village Council is discussing the question of a new water supply, or drainage system, will have a greater significance for them in the future. These men have seen the care taken by the Army authorities in all matters affecting their health while they were soldiers, and they will expect the State to have an equally fatherly regard for their welfare when they become civilians again. Not only will the point of view of men and officers be thus broadened, but their ideas will also be disseminated among all their friends and relations—so that a very large body of public opinion will be reacted upon with a correspondingly great effect.

The lay public are not the only ones who will benefit in this respect through the war; the effect on the medical profession may be just as far-reaching. Many practitioners had little knowledge of public health matters before the war, and others had no practical experience in sanitation. Now it will be different; they will be able to give more authoritative advice on such questions as housing and cleanliness; they will regard the reports of sanitary inspectors with a more critical eye; their widened experience will make them take a keener interest in everything affecting the people's welfare—they will be better doctors. In civil life very few doctors interest themselves actively in public health—that is left entirely to medical officers of health; but on active service every doctor is perforce a sanitarian.

### The Effect on Doctors.

Although many senior combatant officers still fail to appreciate the tremendous importance of this branch of medicine, among the medical men who have worn uniform, at any rate, hygiene has assumed its proper proportions, and is recognised for what it is—something on which the happiness, and, indeed, the very life, of future generations will depend for existence. The general public, including many doctors, has got into the way of taking everything too much for granted. Water, drains, scavenging, all these ordinary everyday necessities are regarded by many just as much a part of their natural environment as the earth and the sky; they give such matters scarcely a thought unless one or other of them "goes wrong." It is not till they have realised what life can be like without such things that they begin to appreciate them at their true worth. Such consequences as follow neglect of Nature's laws have been recognised from time immemorial, but they were often thought to be the result of Divine interposition. The death of the first-born in Egypt, which was quite possibly caused by a widespread epidemic of typhoid, was stated to be a punishment for sin. We also know that our modern "plagues" are only too often the punishment that inevitably follows negligence and carelessness. In this respect the "People's League of Health" should do invaluable service by spreading throughout the country the propaganda of health. The writer had to give a series of lectures, in simple language, on the general principles of hygiene in a scattered and remote rural district some years ago; the interest taken in the subject by the audiences was as significant as their ignorance was appalling. There is plenty of fertile soil; it is for the League to see that the seed sown is of the right sort, so that an abundant harvest of good may be assured.

One of the chief lessons learned during the war has been the use of improvisation. This has been

**Public Health and the War**—*(continued)*.

particularly the case in medicine in general, and in sanitation in particular. It is probable that the effects of this will be felt in civil life. There will be a call for simpler and less costly apparatus. Although it is not suggested that a corrugated iron "incinerator" will replace the type of destructor in use in most of our great towns, there is no doubt that simpler types of destructor would be quite as efficient and less costly. For instance, the "Horsfall"—a well-known type of incinerator—could, and doubtless will be, replaced at many institutions by a brick pattern of a cheaper kind, while the experience of active service has shown that the latter will stand an enormous amount of wear and tear, and, when necessary, can be repaired far more easily and cheaply. Here is a possible way of employing some of our disabled soldiers; many have had considerable experience in constructing and repairing such things on active service, why should they not turn their knowledge and skill to practical advantage? It is hardly likely that chlorination is ever likely to be adopted as a method of purifying water on an extensive scale in the ordinary way—the taste of chlorine would make it unpopular unless some type of subsequent de-chlorination was used; but it might conceivably be useful in villages without a regular supply. At any rate, the stress laid on water purification in the Army should do much towards educating people against the danger of drinking polluted water.

Army life should have done much in teaching the need for greater cleanliness in the preparation and storage of food. It is to be hoped that men coming home will insist on this. At present our shops are a disgrace to us with their supplies of meat, milk, butter, cheese, etc., exposed for sale without any protective covering from dust and flies; it is a wonder that we do not suffer more from fly- and dust-borne diseases, such as enteric fever. In spite of its proved efficacy, it is doubtful if inoculation against this latter disease will be desired by the general public to any greater extent in the near future. This will probably be due partly to the hurry with which inoculation was often necessarily carried out in the field—the malaise and discomfort arising after receiving the full amount in one dose being often quite severe—and partly because it is known that there is not the same danger of becoming infected normally in civil life.

Two practical applications of economy learned in the war might well be introduced into our every-day life; these are the saving of fat and the extraction of solder from old tins. If an average battalion of 1,000 men could get 50 lb. of fat daily from scraps left over and the grease on plates, it is obvious that big establishments such as hotels, restaurants, schools, barracks, etc., could do the same. Even though the demand for glycerin, for the manufacture of explosives, has naturally disappeared, there are other markets for the refined fat, and there should be little difficulty in disposing of it at a profit. As for solder, everyone knows how many tins are used and thrown into the dust-bin in even the smallest households. A solder-extracting machine can be very easily improvised, and solder is very valuable—*verb. sap.*

"Blue Tabs."

